# Higher integrability for singular doubly nonlinear systems

**DOI:** 10.1007/s10231-024-01443-1

**Published:** 2024-04-09

**Authors:** Kristian Moring, Leah Schätzler, Christoph Scheven

**Affiliations:** 1 Fakultät für Mathematik, Universität Duisburg-Essen, Thea-Leymann-Str. 9, 45127 Essen, Germany; 2 Fachbereich Mathematik, Paris-Lodron-Universität Salzburg, Hellbrunner Str. 34, 5020 Salzburg, Austria

**Keywords:** Doubly nonlinear systems, Higher integrability, Gradient estimate, Reverse Hölder inequality, 35B65, 35K40, 35K55

## Abstract

We prove a local higher integrability result for the spatial gradient of weak solutions to doubly nonlinear parabolic systems whose prototype is

$$\begin{aligned} \partial _t \left( |u|^{q-1}u \right) -{{\,\textrm{div}\,}}\left( |Du|^{p-2} Du \right) = {{\,\textrm{div}\,}}\left( |F|^{p-2} F \right) \quad \text { in } \Omega _T:= \Omega \times (0,T) \end{aligned}$$

with parameters $p>1$ and $q>0$ and $\Omega \subset {\mathbb {R}}^n$. In this paper, we are concerned with the ranges $q>1$ and $p>\frac{n(q+1)}{n+q+1}$. A key ingredient in the proof is an intrinsic geometry that takes both the solution *u* and its spatial gradient *Du* into account.

## Introduction

Let $\Omega \subset {\mathbb {R}}^n$, $n \ge 2$, be an open set and $0<T<\infty$. By $\Omega _T:= \Omega \times (0,T)$, we denote the space–time cylinder in ${\mathbb {R}}^{n+1}$. In this paper, we investigate doubly nonlinear systems of the form

**Fig. 1:**
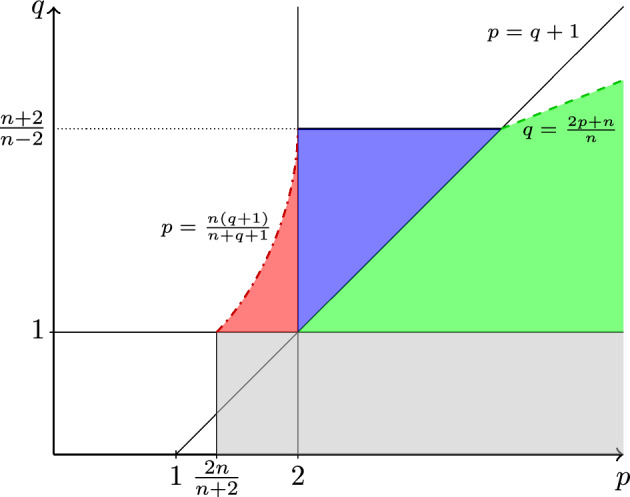
Red, blue, and green areas are the ranges of *p* and *q* covered by Theorem 1.2 (color figure online)

1.1 $$\begin{aligned} \partial _t \left( |u|^{q-1}u \right) -{{\,\textrm{div}\,}}\left( |Du|^{p-2} Du \right) = {{\,\textrm{div}\,}}\left( |F|^{p-2} F \right) \quad \text { in } \Omega _T, \end{aligned}$$

where $q > 0$ and $p >1$. Here, the solution is a map $u :\Omega _T \rightarrow {\mathbb {R}}^N$ for some $N \in {\mathbb {N}}$. Applications include the description of filtration processes, non-Newtonian fluids, glaciers, shallow water flows, and friction-dominated flow in a gas network, see [1, 2, 19, 24, 25, 32] and the references therein. Note that for $q=1$ (1.1) reduces to the parabolic *p*-Laplace system, while for $p=2$ it is the porous medium system (also called fast diffusion system in the singular case $q>1$). Further, the homogeneous equation with $p=q+1$ is often called Trudinger’s equation in the literature. This special case divides the parameter range into two parts where solutions to (1.1) behave differently. In the slow diffusion case $p>q+1$, information propagates with finite speed and solutions may have compact support, whereas in the fast diffusion case $p<q+1$ the speed of propagation is infinite and extinction in finite time is possible. Further, (1.1) becomes singular as $u \rightarrow 0$ and $Du \rightarrow 0$ if $q>1$ and $1<p<2$, respectively, and degenerates as $u \rightarrow 0$ and $Du \rightarrow 0$ if $0<q<1$ and $p>2$, respectively. In this paper, we are interested in the singular range $q>1$ with $p > \frac{n(q+1)}{n+q+1}$. For the precise range that is covered by our main result, see Fig. 1. Moreover, we consider general systems

1.2 $$\begin{aligned} \partial _t \left( |u|^{q-1}u \right) -{{\,\textrm{div}\,}}{\textbf{A}}( x,t,u, Du )= {{\,\textrm{div}\,}}\left( |F|^{p-2} F \right) \quad \text { in } \Omega _T, \end{aligned}$$

where ${\textbf{A}}:\Omega _T\times {\mathbb {R}}^N\times {\mathbb {R}}^{Nn}\rightarrow {\mathbb {R}}^{Nn}$ is a Carathéodory function satisfying

1.3 $$\begin{aligned} \begin{aligned} \left\{ \begin{array}{c} {\textbf{A}}(x,t,u,\xi )\cdot \xi \ge C_o |\xi |^p, \\[5pt] |{\textbf{A}}(x,t,u, \xi )| \le C_1|\xi |^{p-1} \end{array} \right. \end{aligned} \end{aligned}$$

with positive constants $0< C_o \le C_1 < \infty$ for a.e. $(x,t)\in \Omega _T$ and any $(u,\zeta )\in {\mathbb {R}}^n \times {\mathbb {R}}^{Nn}$. Local weak solutions to (1.2) are given by the following definition. In particular, the spatial gradient *Du* lies in the Lebesgue space $L^p(\Omega _T,{\mathbb {R}}^{Nn})$, whose integrability exponent corresponds to the structure conditions (1.3) on ${\textbf{A}}$.

### Definition 1.1

Suppose that the vector field ${\textbf{A}}:\Omega _T \times {\mathbb {R}}^N \times {\mathbb {R}}^{Nn} \rightarrow {\mathbb {R}}^{Nn}$ satisfies (1.3) and $F \in L^p_{{{\,\textrm{loc}\,}}}(\Omega _T,{\mathbb {R}}^{Nn})$. We identify a measurable map $u :\Omega _T \rightarrow {\mathbb {R}}^N$ in the class

$$\begin{aligned} u \in C\big ((0,T);L_{\textrm{loc}}^{q+1}(\Omega ,{\mathbb {R}}^N)\big ) \cap L^p_{\textrm{loc}}\big (0,T; W^{1,p}_{\textrm{loc}}(\Omega ,{\mathbb {R}}^N)\big ) \end{aligned}$$

as a weak solution to (1.2) if and only if

$$\begin{aligned} \iint _{\Omega _T} |u|^{q-1}u\cdot \partial _t \varphi - {\textbf{A}}(x,t,u,Du) \cdot D \varphi \, \,\! \textrm{d}x \,\! \textrm{d}t = \iint _{\Omega _T} |F|^{p-2}F \cdot D \varphi \, \,\! \textrm{d}x \,\! \textrm{d}t \end{aligned}$$

for every $$\varphi \in C_0^\infty (\Omega _T, {\mathbb {R}}^N)$$.

Our main result is that the spatial gradient *Du* of a weak solution to (1.2) is locally integrable to a higher exponent than assumed a priori, provided that *F* is locally integrable to some exponent $\sigma >p$. The precise result is the following.

### Theorem 1.2

Let $1< q < \max \big \{\frac{n+2}{n-2}, \frac{2p}{n}+1 \big \}$, $p > \frac{n(q+1)}{n+q+1}$, $\sigma > p$ and $F \in L^\sigma _{{{\,\textrm{loc}\,}}}(\Omega _T; {\mathbb {R}}^{Nn})$. Then, there exists $\varepsilon _o = \varepsilon _o(n,p,q,C_o,C_1) \in (0,1]$ such that whenever *u* is a weak solution to (1.2) in the sense of Definition 1.1, there holds

$$\begin{aligned} Du \in L^{p(1+\varepsilon _1)}_{{{\,\textrm{loc}\,}}} (\Omega _T; {\mathbb {R}}^{Nn}), \end{aligned}$$

in which $\varepsilon _1 = \min \big \{ \varepsilon _o, \frac{\sigma }{p} - 1 \big \}$. Furthermore, there exists $c = c(n,p,q,C_o,C_1) \ge 1$ such that for every $\varepsilon \in (0,\varepsilon _1]$ and $Q_\varrho = B_{\varrho }(x_o) \times (t_o - \varrho ^{q+1}, t_o + \varrho ^{q+1}) \Subset \Omega _T$ the estimateholds true, where $p^\sharp = \max \{p,q+1 \}$ and

1.4 $$\begin{aligned} d= {\left\{ \begin{array}{ll} \frac{p}{q+1} &{}\quad {}\textrm{if}\, p \ge q+1, \\ \frac{p(q+1)}{p(q+1) +{n}(p-q-1)} &{}\quad {}\textrm{if}\, \frac{{n}(q+1)}{{n}+q+1}< p < q+1. \end{array}\right. } \end{aligned}$$

At this stage, some remarks on the history of the problem are in order. The study of higher integrability was started by Elcrat and Meyers [26], who gave a result for nonlinear elliptic systems. Key ingredients of their proof are a Caccioppoli type inequality and the resulting reverse Hölder inequality, and a version of Gehring’s lemma. The latter was originally used in the context of higher integrability for the Jacobian of quasi-conformal mappings in [13]. For more information, we refer to the monographs [16, Chapter 5, Theorem 1.2] and [18, Theorem 6.7]. The first higher integrability result for parabolic systems is due to Giaquinta and Struwe [17], who were able to treat systems of quadratic growth. However, their technique does not apply to systems of parabolic *p*-Laplace type with general $p\ne 2$. For $p>\frac{2n}{n+2}$, the breakthrough was achieved by Kinnunen and Lewis [22] (see also [23]), whose key idea was to use a suitable intrinsic geometry. More precisely, they considered cylinders of the form $Q_{\varrho ,\lambda ^{2-p}\varrho ^2} := B_\varrho (x_o) \times (t_o-\lambda ^{2-p}\varrho ^2, t_o+\lambda ^{2-p}\varrho ^2)$, where the length of the cylinder depends on the integral average of $|Du|^p$,The concept of intrinsic cylinders has originally been introduced by DiBenedetto and Friedman [11] in connection with Hölder continuity of solutions; see also the monographs [10, 31]. Further, note that the lower bound on *p* in [22] appears naturally in different areas of parabolic regularity theory [10]. In the meantime, [22] has been generalized in several directions, including higher integrability results up to the parabolic boundary [9, 28, 29], and results for higher-order parabolic systems with *p*-growth [3], systems with *p*(*x*, *t*)-growth [4], and most recently parabolic double-phase systems [20, 21].

Despite this progress, higher integrability for the porous medium equation remained open for almost 20 years, since its nonlinearity concerns *u* itself instead of its spatial gradient and is therefore significantly harder to deal with. Then, Gianazza and Schwarzacher [14] succeeded to prove the desired result for non-negative solutions to the degenerate porous medium equation by using intrinsic cylinders that depend on *u* rather than *Du*. The method in [14] relies on the expansion of positivity. Since this tool is only available for non-negative solutions, the approach does not carry over to sign-changing solutions or systems of porous medium type. The case of systems was treated later by Bögelein, Duzaar, Korte and Scheven [6] for the transformed version of (1.2)

$$\begin{aligned} \partial _t u - {{\,\textrm{div}\,}}{\textbf{A}}(x,t,u,D(|u|^{m-1}u)) = {{\,\textrm{div}\,}}F, \end{aligned}$$

where $m = \frac{1}{q} >0$, by using a different intrinsic geometry that also depends on *u* itself. Further, their proof of a reverse Hölder inequality is based on an energy estimate and the so-called gluing lemma, but avoids expansion of positivity. Global higher integrability for degenerate porous medium type systems can be found in [27]. For a local result concerning non-negative solutions in the supercritical singular range $\frac{(n-2)_+}{n+2}<m<1$, we refer to the paper [15] by Gianazza and Schwarzacher, and for sign-changing or vector-valued solutions to the article [8] by Bögelein, Duzaar and Scheven. Analogous to the observation for the singular parabolic *p*-Laplacian above, note that the lower bound $\frac{(n-2)_+}{n+2}$ is natural in the regularity theory for the fast diffusion equation, see [12, Section 6.21].

As a next step, Bögelein, Duzaar, Kinnunen and Scheven [5] proved local higher integrability for the system (1.2) in the homogeneous case $p=q+1$. To this end, they developed a new, elaborate intrinsic geometry that depends on both *u* and *Du*, thus reflecting the doubly nonlinear behavior of the system. The range $\max \big \{1,\frac{2n}{n+2}\big \}<p< \frac{2n}{(n-2)_+}$ of their main result seems unexpected first; however, the lower bound is the natural one for the parabolic *p*-Laplacian, while the upper bound is the same as for the singular porous medium system (note that it can be expressed as $q = p-1 < \frac{n+2}{(n-2)_+}$). For $N=1$, non-negative solutions and $F \equiv 0$, Saari and Schwarzacher [30] were able to remove the upper bound for all dimensions $n \in {\mathbb {N}}$. Finally, the range $0<q<1$ and $\frac{2n}{n+2} < p$ of (1.2), i.e., the degenerate case with respect to *u*, has been dealt with by Bögelein, Duzaar and Scheven in [7]. The range covered by [7] corresponds to the gray area in Fig. 1.

The goal of the present paper is to treat the singular range $q>1$ and thus close the gap in the higher integrability theory for (1.2). The overall strategy is similar to the one in [7]. However, there is a crucial difference in the chosen intrinsic geometry. While scaling in the time variable is appropriate in the degenerate case, the technique seems to require a different scaling in the singular case. Thus, we work with a scaling both in the spatial and time variables. Namely, throughout the article we consider cylinders of the form

$$\begin{aligned} Q_\varrho ^{(\lambda ,\theta )}(x_o,t_o):= B_{\theta ^\frac{1-q}{1+q} \varrho }(x_o) \times (t_o - \lambda ^{2-p} \varrho ^{1+q}, t_o + \lambda ^{2-p} \varrho ^{1+q}) \end{aligned}$$

with positive factors $\lambda ,\theta$ and $(x_o,t_o) \in \Omega _T$. We collect technical lemmas, energy estimates and the gluing lemma for such cylinders in Sect. 2. In particular, the latter two have already been proved in [7] for all $p>1$ and $q>0$. Now, the idea is to select $\lambda$ and $\theta$ such that1.5in order to obtain intrinsic cylinders. However, due to some complications related to their construction, we also need to take so-called $\theta$-subintrinsic cylinders into account, where only the inequality "$\gtrsim$" is satisfied in (1.5)_2_. More precisely, we can construct cylinders in such a way that they are either $\theta$-intrinsic in the sense of (1.5)_2_ or that they are $\theta$-subintrinsic and satisfy $\theta \lesssim \lambda$, see (3.3). We call the latter case $\theta$-singular because it means that *u* is in a certain sense small compared to its oscillation, and the differential equation becomes singular if |*u*| becomes small. In both cases, sophisticated arguments are necessary to prove parabolic Sobolev–Poincaré type inequalities for all relevant cylinders. This is done in the regime $\frac{n(q+1)}{n+q+1} < p \le q+1$ in Sect. 3 and in the range $2< q+1 < p$ in Sect. 4. Reverse Hölder inequalities in the same types of cylinders are shown for the whole range $q>1$ and $\frac{n(q+1)}{n+q+1} < p$ in Sect. 5. The lower bound on *p* appearing in the proof of these vital tools and thus restricting the red area of admissible parameters in Fig. 1 is natural in the regularity theory of the doubly nonlinear Eq. (1.1). Finally, the proof of Theorem 1.2 is found in Sect. 6. To this end, we start with a given non-intrinsic cylinder $Q_{2R} \Subset \Omega _T$ and first focus on the second relation in (1.5) in Sect. 6.1. This is the step where, in the case $n\ge 3$, the conditions $q<\frac{n+2}{n-2}$ for $p<q+1$ and $q<\frac{2p}{n}+1$ for $p>q+1$ restricting the blue and green parameter areas in Fig. 1 come into play. These conditions are consistent with the bounds $q <\frac{n+2}{n-2}$ for the singular porous medium system in [8] and $q+1=p<\frac{2n}{n-2}$ for the homogeneous doubly nonlinear system in [5]. Even in the latter special case, it remains an interesting open problem to remove this condition in the case of systems.

Ideally, we would like to choose $\theta$ in dependence on given parameters $\lambda$ and $\varrho$ such that $\varrho \mapsto \theta$ (with fixed $\lambda$) is non-increasing and that $Q_\varrho ^{(\lambda ,\theta )}\subset Q_{2R}$ satisfies (1.5)_2_. The reason that it is only possible to obtain $\theta$-subintrinsic cylinders is the so-called sunrise construction that is used to ensure the monotonicity of $\varrho \mapsto \theta$. Next, we prove a Vitali-type covering property for the relevant cylinders in Sect. 6.2. In Sect. 6.3, for given $\lambda$ we use a stopping time argument to fix the radius of our (sub)-intrinsic cylinders (and thus the parameter $\theta$ according to the first step) such that also the first relation in (1.5) is satisfied. Applying the results of Sect. 5, we show that a suitable reverse Hölder inequality holds in Sect. 6.4. Finally, we sketch standard arguments that finish the proof in Sect. 6.5.

## Preliminaries

We write $z_o = (x_o,t_o) \in {\mathbb {R}}^n \times {\mathbb {R}}$ and use space–time cylinders of the form

$$\begin{aligned} Q_\varrho ^{(\lambda , \theta )}(z_o) = B_\varrho ^{(\theta )}(x_o) \times \Lambda _\varrho ^{(\lambda )}(t_o), \end{aligned}$$

where

$$\begin{aligned} B_\varrho ^{(\theta )}(x_o) = \left\{ x\in {\mathbb {R}}^n: |x-x_o| < \theta ^\frac{1-q}{1+q} \varrho \right\} , \end{aligned}$$

and

$$\begin{aligned} \Lambda ^{(\lambda )}_\varrho (t_o) = \left( t_o - \lambda ^{2-p} \varrho ^{1+q}, t_o + \lambda ^{2-p} \varrho ^{1+q} \right) , \end{aligned}$$

with parameters $\theta , \lambda > 0$. If $\lambda = \theta = 1$, we use the simpler notation

$$\begin{aligned} Q_\varrho (z_o):= Q_\varrho ^{(1,1)}(z_o). \end{aligned}$$

For the mean value of a function $u\in L^1(Q)$ over a cylinder $Q=B\times \Lambda \subset {\mathbb {R}}^{n}\times {\mathbb {R}}$ of finite positive measure, we writeand similarly,for the slice-wise means, provided $u(\cdot ,t)\in L^1(B)$. In the particular cases $Q=Q_\varrho ^{(\lambda , \theta )}(z_o)$ and $B=B_\varrho ^{(\theta )}(x_o)$, we also write

$$\begin{aligned} (u)_{z_o;\varrho }^{(\lambda , \theta )}:= (u)_{\varrho }^{(\lambda , \theta )}:= (u)_Q \qquad \text{ and }\qquad (u)_{x_o;\varrho }^{(\theta )}(t):= (u)_{\varrho }^{(\theta )}(t):= (u)_B(t). \end{aligned}$$

For the power of a vector $u\in {\mathbb {R}}^N$ to an exponent $\alpha >0$, we write

$$\begin{aligned} {\varvec{u}}^\alpha :=|u|^{\alpha -1}u, \end{aligned}$$

where we interpret the right-hand side as zero if $u=0$.

Next we state a useful iteration lemma that can be obtained by a change of variables in [18, Lemma 6.1].

### Lemma 2.1

Let $0<\vartheta <1$, $A,C\ge 0$ and $\alpha ,\beta >0$. Then, there exists a constant $c=c(\alpha ,\beta ,\vartheta )$ such that there holds: For any $0<r<\varrho$ and any nonnegative bounded function $\phi :[r,\varrho ] \rightarrow {\mathbb {R}}_{\ge 0}$ satisfying

$$\begin{aligned} \phi (t) \le \vartheta \phi (s) +A(s^\alpha -t^\alpha )^{-\beta }+C \quad \text { for all } r\le t <s \le \varrho , \end{aligned}$$

we have

$$\begin{aligned} \phi (r) \le c \left[ A(\varrho ^\alpha -r^\alpha )^{-\beta }+C \right] . \end{aligned}$$

Using the arguments of [18, Lemma 8.3], the following lemma can be deduced.

### Lemma 2.2

For every $\alpha >0$, there exists a constant $c=c(\alpha )$ such that, for all $a,b\in {\mathbb {R}}^N$, $N\in {\mathbb {N}}$, we have

$$\begin{aligned} \tfrac{1}{c}\big |{{\varvec{b}}^{{\alpha }}} - {{\varvec{a}}^{{\alpha }}}\big | \le \big (|a| + |b|\big )^{\alpha -1}|b-a| \le c \big |{{\varvec{b}}^{{\alpha }}} - {{\varvec{a}}^{{\alpha }}}\big |. \end{aligned}$$

In the case $\alpha \ge 1$, the preceding lemma immediately implies the following elementary estimate.

### Lemma 2.3

For every $\alpha \ge 1$, there exists a constant $c=c(\alpha )$ such that, for all $a,b\in {\mathbb {R}}^N$, $N\in {\mathbb {N}}$, we have

$$\begin{aligned} |b-a|^\alpha \le c\big |{{\varvec{b}}^{{\alpha }}} - {{\varvec{a}}^{{\alpha }}}\big |. \end{aligned}$$

For the proof of the following statement on the quasi-minimality of the mean value, we refer to [5, Lemma 3.5].

### Lemma 2.4

Let $p \ge 1$ and $\alpha \ge \frac{1}{p}$. There exists a constant $c = c(\alpha ,p)$ such that whenever $A \subset B \subset {\mathbb {R}}^k$, $k \in {\mathbb {N}}$ holds for bounded sets *A* and *B* of positive measure, then for every $u \in L^{\alpha p}(B,{\mathbb {R}}^N)$ and $a \in {\mathbb {R}}^N$ there holds

Next, we recall the Gagliardo–Nirenberg inequality.

### Lemma 2.5

Let $1 \le p,q, r < \infty$ and $\vartheta \in (0,1)$ such that $-\frac{n}{p} \le \vartheta (1- \frac{n}{q}) - (1-\vartheta ) \frac{n}{r}$. Then, there exists a constant $c = c(n,p)$ such that for any ball $B_\varrho (x_o) \subset {\mathbb {R}}^n$ with $\varrho > 0$ and any function $u \in W^{1,q}(B_\varrho (x_o))$ we have

Finally, the proof of the following two lemmas can be found in [7]. We note that in [7], a slightly different definition of intrinsic cylinders has been used. In order to obtain the following statements, we replace the radii $\varrho ,r$ in [7] by $\theta ^{\frac{1-q}{1+q}}\varrho ,\theta ^{\frac{1-q}{1+q}} r$. We start with an energy estimate for solutions of (1.2).

### Lemma 2.6

([7, Lemma 3.1]) Let $p>1$, $q > 0$ and *u* be a weak solution to (1.2) where the vector field ${\textbf{A}}$ satisfies (1.3). Then, there exists a constant $c=c(p,q,C_o,C_1)$ such that on every cylinder $Q_\varrho ^{(\lambda ,\theta )}(z_o)\Subset \Omega _T$ with $\varrho >0$ and $\lambda , \theta >0$ and for any $r\in [\varrho /2,\varrho )$ and all $a \in {\mathbb {R}}^N$ the following energy estimate2.1holds true.

Then we state the gluing lemma.

### Lemma 2.7

([7, Lemma 3.2]) Let $p>1$, $q > 0$ and *u* be a weak solution to (1.2) where the vector field ${\textbf{A}}$ satisfies (1.3). Then, there exists a constant $c=c(C_1)$ such that on every cylinder $Q_{\varrho }^{(\lambda ,\theta )}(z_o)\Subset \Omega _T$ with $\varrho >0$ and $\lambda , \theta >0$ there exists ${\hat{\varrho }} \in \left[ \frac{\varrho }{2},\varrho \right]$ such that for all $t_1,t_2 \in \Lambda _\varrho ^{(\lambda )}(t_o)$ there holds

## Parabolic Sobolev–Poincaré type inequalities in case $q+1 \ge p$

The goal of this section is to prove Sobolev–Poincaré inequalities that bound the right-hand side of the energy estimate (2.1) from above. It turns out that different strategies are required for the cases $q+1\ge p$ and $q+1<p$. Therefore, we only consider the first case here and postpone the second one to the next section.

We use $\lambda$-intrinsic3.1$\theta$-intrinsic3.2scalings, in which $p^\sharp = \max \{p,q+1\}$. However, for the cylinders constructed in Sect. 6.1, we are not able to prove the $\theta$-intrinsic scaling in every case. In general, we can only prove the first of the two inequalities in (3.2), which we refer to as $\theta$-subintrinsic scaling. In Sect. 6.4, we will show that the cylinders used in the proof either satisfy the $\theta$-intrinsic scaling (3.2) or a scaling of the form3.3We call this scaling $\theta$-singular because it means that the solution is in a certain sense small compared to its oscillation, in which case differential Eq. (1.2) becomes singular.

For now, we suppose that $q+1 \ge p$. Then (3.2) reads as3.4and (3.3) as3.5We start with a Sobolev–Poincaré type estimate for the second term appearing on the right-hand side of the energy estimate from Lemma 2.6.

### Lemma 3.1

Suppose that $q > 1$, $\frac{n(q+1)}{n+q+1}< p \le q+1$, and that *u* is a weak solution to (1.2), under assumption (1.3). Moreover, we consider a cylinder $Q_{2\varrho }^{(\lambda ,\theta )}(z_o) \Subset \Omega _T$ and assume that (3.1) is satisfied together with either (3.4) or (3.5). Then the following Sobolev–Poincaré inequality holds:where $\max \left\{ \frac{n(q+1)}{p(n+q+1)} , \frac{p-1}{p} \right\} \le \nu \le 1$ and $a = (u)_{z_o;\varrho }^{(\theta ,\lambda )}$. The preceding estimate holds for an arbitrary $\varepsilon \in (0,1)$ with a constant $c = c(n,p,q,C_1,C_\theta ,C_\lambda )> 0$ and $\beta = \beta (n,p,q) > 0$.

### Proof

Since the cylinder is fixed throughout the proof, we use the more compact notations $Q:=Q_\varrho ^{(\theta ,\lambda )}(z_o)$, $B:=B_\varrho ^{(\theta )}(x_o)$ and $\Lambda :=\Lambda _\varrho ^{(\lambda )}(t_o)$. Furthermore, with the radius ${\hat{\varrho }}\in \left[ \frac{\varrho }{2},\varrho \right]$ provided by Lemma 2.7, we write ${\widehat{B}}:=B_{{\hat{\varrho }}}^{(\theta )}(x_o)$ and $\widehat{Q}:={\widehat{B}}\times \Lambda$. Using first Lemma 2.4 with $\alpha =\frac{q+1}{2}$ and $p=2$ and then the triangle inequality, we estimate3.6We use Lemma 2.4 with $\alpha =\frac{q+1}{2q}$ and $p=2$ to estimate3.7In the last inequality, we also used Young’s inequality with exponents $\frac{n+2}{2}$ and $\frac{n+2}{n}$. Observe that Lemma 2.2 and Hölder’s inequality implyBy applying Hölder inequality in the time integral with exponents $\frac{n+2}{n}\cdot \frac{q+1}{q-1}$ and $\frac{n+2}{2}\cdot \frac{q+1}{n+q+1}$, we obtainBy $\theta$-subintrinsic scalingand by Sobolev inequality, we haveWe combine the estimates and obtain3.8The last estimate follows from Hölder’s inequality, since $\nu p\ge \frac{n(q+1)}{n+q+1}$. In the case $p<2$, we use the $\lambda$-subintrinsic scaling (3.1)_1_ and Hölder’s inequality, which yields the boundwhile in the case $p\ge 2$, we use Young’s inequality. In both cases, we observe that (3.8) implieswhere the term $\varepsilon \lambda ^p$ can be omitted in the case $p<2$. Here and in the remainder of the proof, we write $\beta$ for a positive universal constant that depends at most on *n*, *p* and *q*. Bounding the right-hand side by the $\lambda$-superintrinsic scaling (3.1)_2_ and using the resulting estimate to bound the right-hand side of (3.7) from above, we deduce3.9Then let us turn our attention to the term $\textrm{II}$. We apply in turn Lemma 2.3 with $\alpha =\frac{2q}{q+1}\ge 1$ and then Lemma 2.7 to get3.10In the case (3.4), we estimatewhere we used Lemma 2.4 with $\alpha =\frac{q+1}{2q}$ and $p=2$ in the last step. We use this to estimate

$$\begin{aligned} \textrm{II} = \frac{\theta ^{\frac{2(q-1)}{q+1}}}{\theta ^{\frac{2(q-1)}{q+1}}} \textrm{II} \le \textrm{II}_1 + \textrm{II}_2, \end{aligned}$$

where we denotedand

$$\begin{aligned} \textrm{II}_2 := \frac{c|({\varvec{u}}^q)_{{\widehat{Q}}}|^\frac{q-1}{q}}{\theta ^\frac{2(q-1)}{q+1} \varrho ^{q-1}} \cdot \textrm{II}. \end{aligned}$$

For the estimate of $\textrm{II}_1$, we use in turn (3.10) the $\theta$-subintrinsic scaling and then Young’s inequality with exponents $\frac{2q}{q-1}$ and $\frac{2q}{q+1}$, with the resultUsing the definition of $\textrm{II}$ and Lemma 2.3, we also haveIn the last step, we used Lemma 2.7. We combine the two preceding estimates to3.11In order to estimate the last term further, we distinguish between the cases $p \ge 2$ and $p<2$. In the first case, we use the $\lambda$-intrinsic scaling (3.1), which impliesIn the case $p<2$, we apply Young’s inequality with exponents $\frac{p}{2-p}$ and $\frac{p}{2(p-1)}$. In both cases, we deduce that (3.11) implies3.12for every $\varepsilon \in (0,1)$. This completes the estimate of $\textrm{II}$ in the case (3.4). On the other hand, in the case (3.5) we haveIn the last step, we used (3.1). Inserting this estimate into (3.10), we obtainIf $q+1>p$, we apply Young’s inequality with exponents $\frac{pq}{q+1-p}$ and $\frac{pq}{(p-1)(q+1)}$ and arrive atIn the borderline case $q+1=p$, the same estimate is immediate. Consequently, the bound (3.12) for $\textrm{II}$ holds true in every case considered in the lemma. Combining this with estimate (3.9) of $\textrm{I}$ and recalling the definition of $\textrm{I}$ and $\textrm{II}$ in (3.6), we deduceWe reabsorb the first term on the right-hand side into the left-hand side and estimate the term $\lambda ^p$ by the $\lambda$-intrinsic scaling (3.1). This yields the asserted estimate after replacing $\varepsilon$ by $\frac{\varepsilon }{c}$. $\square$

Next, we give an auxiliary result that will be needed in the proof of the second Sobolev–Poincaré inequality.

### Lemma 3.2

Let $q > 1$, $\frac{n(q+1)}{n+q+1} < p \le q+1$ and assume that $Q_{2\varrho }^{(\lambda ,\theta )}(z_o) \Subset \Omega _T$ and that the $\lambda$- and $\theta$-subintrinsic scaling properties (3.1)_1_ and (3.4)_1_ are satisfied. Then, there exists a constant $c > 0$ depending on $n,p,q,C_\theta$ and $C_\lambda$ such that for every function $u\in L_{\textrm{loc}}^p(0,T;W_{\textrm{loc}}^{1,p}(\Omega ,{\mathbb {R}}^N))\cap L^\infty _{\textrm{loc}}(0,T;L_{\textrm{loc}}^{q+1}(\Omega ,{\mathbb {R}}^N))$, we havefor every $\nu \in \left[ \frac{n(q+1)}{p(n+q+1)},1\right]$, every ${\hat{\varrho }}\in \left[ \frac{\varrho }{2},\varrho \right]$ and every $a\in {\mathbb {R}}^N$. In particular, we have

### Proof

As in the preceding proof, we abbreviate $Q:=Q_\varrho ^{(\lambda ,\theta )}(z_o)$, $B:=B_\varrho ^{(\theta )}(x_o)$, ${\widehat{B}}:=B_{{\hat{\varrho }}}^{(\theta )}(x_o)$ and $\Lambda :=\Lambda _\varrho ^{(\lambda )}(t_o)$. First, we apply Lemma 2.4 with $\alpha =\frac{1}{q}$ and $p=q+1$ to exchange the mean value of ${\varvec{u}}^q$ by the mean value of *u*. Then, we note that the fact $\nu \ge \frac{n(q+1)}{p(n+q+1)}$ allows us to use the Gagliardo–Nirenberg inequality from Lemma 2.5 with the parameters $(p,q,r,\vartheta )$ replaced by $(q+1,\nu p,q+1,\frac{\nu p}{q+1})$. Finally, we apply Poincaré’s inequality slicewise. In this way, we obtainIn the last step, we applied Lemma 2.4 again. We use assumption (3.4)_1_ in order to bound the negative power of $\theta$ appearing on the right-hand side from above. In this way, we obtainBy absorbing the first integral on the right-hand side into the left and taking both sides to the power $\frac{2(q+1)}{2(q+1)+\nu p(q-1)}$, we deduce the first asserted estimate. The second assertion follows by choosing $\nu =1$ and using (3.1)_1_. $\square$

Now we are in a position to prove a Sobolev–Poincaré inequality for the first term on the right-hand side of the energy estimate (2.1).

### Lemma 3.3

Suppose that $q > 1$, $\frac{n(q+1)}{n+q+1}<p \le q+1$, and that *u* is a weak solution to (1.2), where assumption (1.3) is satisfied. Moreover, we consider a cylinder $Q_{2\varrho }^{(\lambda ,\theta )}(z_o) \Subset \Omega _T$ and assume that the $\lambda$-intrinsic coupling (3.1) and additionally, property (3.4) or (3.5) are satisfied. Then the following Sobolev–Poincaré inequality holds:where $\max \left\{ \frac{n(q+1)}{p(n+q+1)} , \frac{p-1}{p},\frac{n}{n+2},\frac{n}{n+2}\left( 1+\frac{2}{p}-\frac{2}{q}\right) \right\} \le \nu \le 1$ and $a = (u)_{z_o;\varrho }^{(\theta ,\lambda )}$. The preceding estimate holds for an arbitrary $\varepsilon \in (0,1)$ with a constant $c = c(n,p,q,C_1,C_\theta ,C_\lambda )> 0$ and $\beta = \beta (n,p,q)>0$.

### Proof

We continue to use the notations $Q,{\widehat{Q}},B,{\widehat{B}}$ and $\Lambda$ introduced in the preceding proofs. We begin with two easy cases, in which the assertion can be deduced from Lemma 3.1.

*Case 1: The *$\theta$-*singular case (*3.5*).* In this case, assumptions (3.5) and (3.1) imply $\theta \le c\lambda$. Moreover, we use Hölder’s inequality, Lemma 2.3 with $\alpha =\frac{q+1}{2}$, and finally, Young’s inequality with exponents $\frac{q+1}{q+1-p}$ and $\frac{q+1}{p}$. In this way, we obtain the boundAgain, we write $\beta$ for a positive universal constant that depends at most on *n*, *p* and *q*. At this stage, the claim follows by estimating the last term with the help of Lemma 3.1.

*Case 2: The *$\theta$-*intrinsic case (*3.4*) with* $p\le 2$. As a consequence of (3.4) we haveUsing this together with Hölder’s inequality, we inferWe estimate the first term on the right-hand side by Lemma 2.3 with $\alpha =\frac{q+1}{2}$ and the second term by Lemma 2.2 with the same value of $\alpha$. In this way, we getThe last estimate follows from Hölder’s inequality, since $p\le 2$. If $p < 2$, we may directly use Young’s inequality with exponents $\frac{2}{2-p}$ and $\frac{2}{p}$, which results in the estimatefor every $\varepsilon \in (0,1)$. In the case $p=2$, this is an immediate consequence of the preceding inequality. Now, the asserted estimate again follows by applying Lemma 3.1 to the last integral.

Now we turn our attention to the final case, which turns out to be much more involved.

*Case 3: The *$\theta$-*intrinsic case (*3.4*) with* $p>2$. By using triangle inequality and Lemma 2.4 with $\alpha =1$, we writeThe $\theta$-superintrinsic scaling (3.4)_2_ impliesWe use this to estimate the term $\textrm{I}$ and twice apply Hölder’s inequality in the space integral, denoting $\sigma = \max \{p,q\}$. Afterward, we apply Lemma 2.4, once with $\alpha =\frac{1}{q}$ and $p=\sigma$ and once with $\alpha =\frac{1}{q}$ and $p=q+1$. Note that in particular the first application is possible since $\sigma \ge q$. This procedure leads to the estimateBy using Lemma 2.5 with $(p,q,r,\vartheta )$ replaced by $(\sigma ,\nu p,2,\nu )$, which is possible since $\nu \ge \frac{n}{n+2} \max \left\{ 1, 1+\frac{2}{p} - \frac{2}{q} \right\}$, we have3.13In the next step, we use Poincaré’s inequality slice-wise and rearrange the terms. Then, we note that the $\theta$-subintrinsic scaling (3.4)_1_ implies $\left( \frac{|a|}{\varrho }\right) ^{q+1}\le c\theta ^2$. For the estimate of the $\sup$-term, we use Lemma 2.4 with $\alpha =1$ and $p=2$, and then Lemma 2.2 with the parameter $\alpha =\frac{q+1}{2}$. This leads to the estimateSince $\nu \ge \frac{p-1}{p}$, we may use Young’s inequality with exponents $\frac{2}{(1-\nu )p}$ and $\frac{2}{2 - (1-\nu )p}$ to getBy using the $\lambda$-subintrinsic scaling (3.1)_1_, which implies3.14together with the fact $p>2$, we arrive at the estimate3.15Next, we estimate the term $\textrm{I}_2$. Since $p > \frac{n(q+1)}{n+q+1}$, the Sobolev–Poincaré inequality implies3.16In the last step, we used (3.1). Furthermore, since *Q* is $\theta$-subintrinsic in the sense of (3.4)_1_, we haveEstimating the right-hand side by (3.16), we observe that the powers of $\theta$ cancel each other out. Therefore, we obtain the bound3.17In order to estimate $\textrm{I}_2$, we apply the triangle inequality and use (3.17) in the first of the resulting terms and (3.16) in the second. This leads to the boundFor the estimate of the first term, we use Young’s inequality with exponents $\frac{q+1}{q-1}$ and $\frac{q+1}{2}$ and then Lemma 3.2, which yields the boundSince $2<p\le q+1$, the power of $\lambda$ in the last line is negative. Therefore, we can use the $\lambda$-subintrinsic scaling (3.1)_1_ in the form of (3.14) to estimate the power of $\lambda$ from above. This leads to the boundSince $\nu p\ge p-1>p-2$, the exponent of the $\sup$-term is smaller than one, and it is positive. Moreover, both exponents outside the round brackets add up to one. Therefore, another application of Young’s inequality yields3.18For the estimate of $\textrm{I}_{2,2}$, we use Lemma 2.3 with $\alpha =q$ and then Lemma 2.7, which implies3.19Note that we can assumesince otherwise, the assertion of the lemma clearly holds, because (3.4)_1_ implies that the left-hand side of the asserted estimate is bounded by $c\theta ^p$. Using this observation in order to bound the negative powers of $\theta$ in the preceding estimate, we arrive atIn case $2q + (2-p)(q-1) < 0$, we use the $\lambda$-subintrinsic scaling (3.1)_1_ and obtainIf $2q + (2-p)(q-1) = 0$, this estimate is identical to the preceding one. In the remaining case, by observing that $\frac{2q + (2-p)(q-1)}{2q} < 1$, we use Young’s inequality with exponents $\frac{2q}{2q + (2-p)(q-1)}$ and $\frac{2q}{(p-2)(q-1)}$ to obtaincompleting the treatment of the term $\textrm{I}_{2,2}$. Combining this result with (3.15) and (3.18), using Hölder’s inequality and Lemma 2.3, we infer the bound3.20By the $\theta$-superintrinsic scaling (3.4)_2_, we havewhere we abbreviated ${{\hat{a}}} = [({\varvec{u}}^q)_{\widehat{Q}}]^\frac{1}{q}$. Using this for the estimate of $\textrm{II}$, we obtainFor the first term, we use in turn Lemma 2.2 with $\alpha =q$, the gluing lemma (Lemma 2.7), the $\lambda$-subintrinsic scaling (3.1)_1_, and then Hölder’s inequality to get3.21For the term $\textrm{II}_3$, we use Lemma 2.3 with $\alpha =q$ and then Hölder’s inequality to estimateby using also the fact $\frac{q+1}{q}\le 2<p$. Now we proceed exactly as for the estimate of $\textrm{II}_1$ and arrive at the boundFor the term $\textrm{II}_2$, we divide the power of the second term as $p \frac{q-1}{q+1} = \frac{p(q-1)^2}{2q(q+1)}+\frac{p(q-1)}{2q}$ and estimate the first part using the $\theta$-subintrinsic scaling (3.4)_1_. For the last integral in $\textrm{II}_2$, we apply Lemma 2.3 with $\alpha =q$. The resulting integrals are then estimated by Lemma 3.2 and Lemma 2.7, respectively. This yieldsObserve that $\theta$ will cancel out on the right-hand side. Subsequently, we use Young’s inequality with exponents *q* and $\frac{q}{q-1}$ and obtainFor the first term, we use Young’s inequality with exponents $\frac{2(q+1)+p(q-1)}{2(q+1) + p(p-2)}$ and $\frac{2(q+1) + p(q-1)}{p(q+1-p)}$ (observe that these exponents are $> 1$ in case $2< p < q+1$). For the last term, we use the $\lambda$-subintrinsic scaling (3.1)_1_ and the fact $p>2$ to deduceCollecting the estimates and applying Hölder’s inequality and Lemma 2.3, we arrive at the boundAs stated in (3.20), the term $\textrm{I}$ is bounded by exactly the same quantities. Therefore, the asserted estimate follows by bounding $\lambda ^p$ by means of the $\lambda$-intrinsic scaling (3.1). $\square$

## Parabolic Sobolev–Poincaré type inequalities in case $q+1 < p$

In this section, we prove versions of the Sobolev–Poincaré type inequalities from the preceding section for the missing case $q+1<p$. In this case, the $\theta$-intrinsic scaling (3.2) reads as4.1and the $\theta$-singular scaling (3.3) becomes4.2We start with an auxiliary estimate that will be needed for the estimate of the first Sobolev–Poincaré inequality.

### Lemma 4.1

Let $p> q+1 > 2$ and assume that $Q_{2\varrho }^{(\lambda ,\theta )}(z_o) \Subset \Omega _T$ and that the $\lambda$- and $\theta$-subintrinsic scaling properties (3.1)_1_ and (4.1)_1_ are satisfied. Then, there exists a constant $c > 0$ depending on $n,p,q,C_\theta$ and $C_\lambda$ such that for every function $u\in L_{\textrm{loc}}^p(0,T;W_{\textrm{loc}}^{1,p}(\Omega ,{\mathbb {R}}^N))\cap L^\infty _{\textrm{loc}}(0,T;L_{\textrm{loc}}^{q+1}(\Omega ,{\mathbb {R}}^N))$, we havefor every $\nu \in \left[ \frac{n}{n+q+1},1\right]$ and every $a\in {\mathbb {R}}^N$. In particular, we have

### Proof

As in the preceding section, we abbreviate $Q:=Q_\varrho ^{(\lambda ,\theta )}(z_o)$, $B:=B_\varrho ^{(\theta )}(x_o)$, ${\widehat{B}}:=B_{{\hat{\varrho }}}^{(\theta )}(x_o)$ and $\Lambda :=\Lambda _\varrho ^{(\lambda )}(t_o)$. We note that the fact $\nu \ge \frac{n}{n+q+1}$ allows us to use the Gagliardo–Nirenberg inequality from Lemma 2.5 with the parameters $(p,q,r,\vartheta )$ replaced by $(p,\nu p,q+1,\nu )$. Finally, we apply Poincaré’s inequality slicewise. In this way, we obtainIn the last step, we applied Lemma 2.4. We use assumption (4.1)_1_ in order to bound the negative power of $\theta$ appearing on the right-hand side from above. In this way, we obtainBy absorbing the first integral on the right-hand side into the left and taking both sides to the power $\frac{2}{2+\nu (q-1)}$, we deduce the first asserted estimate. The second assertion follows by choosing $\nu =1$ and using (3.1)_1_. $\square$

Next, we prove a Sobolev–Poincaré type inequality for the first term on the right-hand side of the energy estimate (2.1).

### Lemma 4.2

Suppose that $p>q+1 > 2$ and that *u* is a weak solution to (1.2), under assumption (1.3). Moreover, we consider a cylinder $Q_{2\varrho }^{(\lambda ,\theta )}(z_o) \Subset \Omega _T$ and assume that the $\lambda$-intrinsic coupling (3.1) and additionally property (4.1) or (4.2) are satisfied. Then the following Sobolev–Poincaré inequality holds:where $\max \left\{ \frac{p-1}{p},\frac{n}{n+2} \right\} \le \nu \le 1$ and $a = (u)_{z_o;\varrho }^{(\theta ,\lambda )}$. The preceding estimate holds for any $\varepsilon \in (0,1)$ with a constant $c = c(n,p,q,C_1,C_\theta ,C_\lambda )> 0$ and $\beta = \beta (n,p,q)>0$.

### Proof

We continue to use the notations $Q,{\widehat{Q}},B,{\widehat{B}}$ and $\Lambda$ introduced in the preceding proofs. First observe that $p > q+1$ implies $p > 2$. We distinguish between the cases (4.2) and (4.1).

*Case 1: The *$\theta$-*singular case (*4.2*).* We use Lemma 2.4 and the triangle inequality to estimatewith ${{\hat{a}}} = [({\varvec{u}}^q)_{{\widehat{Q}}}]^\frac{1}{q}$. For the first term, we use Lemmas 2.4 and 2.5 with $(p,q,r,\vartheta )=(p,\nu p, q+1,\nu )$ to obtainObserve that $\nu \ge \frac{n}{n+2} > \frac{n}{n+q+1}$ such that Lemma 2.5 is applicable. Now we use (4.2) and (3.1) which implyThen we apply Young’s inequality with the power $\frac{q+1}{(1-\nu )p}$ and its conjugate, which are greater than one since $\nu \ge \frac{p-1}{p}$. This concludes the claim for the first term.

For the second term, we use Lemma 2.7 and deducesince assumptions (4.2) and (3.1) imply $\theta \le c\lambda$ and $p > q+1$, which concludes the proof in this case.

*Case 2: The *$\theta$-*intrinsic case (*4.1*).* By using triangle inequality and Lemma 2.4 with $\alpha =1$, we writeThe $\theta$-superintrinsic scaling (4.1)_2_ impliesWe use this to estimate the term $\textrm{I}$ and apply Lemma 2.4 with $\alpha =\frac{1}{q}$ and *p*. Note that the application is possible since $p> q+1>q$. This procedure leads to the estimatewhere we abbreviated ${{\hat{a}}} = [({\varvec{u}}^q)_{\widehat{Q}}]^\frac{1}{q}$. By using Lemma 2.5 with $(p,q,r,\vartheta )$ replaced by $(p,\nu p,2,\nu )$, which is possible since $\nu \ge \frac{n}{n+2}$, we haveThis is exactly the same estimate as (3.13) in the proof of Lemma 3.3. Therefore, we can repeat the arguments leading to (3.15) and obtainNext, we estimate the term $\textrm{I}_2$. Observe that Lemma 2.4 impliesFurthermore, by applying Lemma 4.1 and (3.1)_1_ we haveSince $\nu \ge \frac{p-1}{p}$, the exponents outside the round brackets are less than one, and furthermore, they add up to one. Thus, we may use Young’s inequality which completes the treatment of the term $\textrm{I}_2$.

Then, we consider the term $\textrm{I}_3$. By using Lemma 2.7 for the first term and Poincaré inequality for the second, we obtainThis corresponds to estimate (3.19) for the term $\textrm{I}_{2,2}$ in the proof of Lemma 3.3. Therefore, arguing as after estimate (3.19), we deduceBy the $\theta$-superintrinsic scaling (4.1)_2_, we havewhere ${{\hat{a}}} = [({\varvec{u}}^q)_{\widehat{Q}}]^\frac{1}{q}$. Using this for the estimate of $\textrm{II}$, we obtainFor the first term, we use Lemma 2.2, which implieswhile the third term is estimated with the help of Lemma 2.3 and Hölder’s inequality, which givesTherefore, both terms can be estimated as in (3.21), with the resultFor the term $\textrm{II}_2$, we estimate the first part using the $\theta$-subintrinsic scaling (4.1)_1_ and for the last integral we apply Lemma 2.3 with $\alpha =q$. The resulting integrals are then estimated by Lemma 4.1 and Lemma 2.7, respectively. This yieldswhere we also used Young’s inequality with exponents $\frac{q}{q+1-p}$ and $\frac{q}{p-1}$ on the last line. Thus, the claim follows. $\square$

Finally, we state the Sobolev–Poincaré inequality for the second term on the right-hand side of (2.1). It turns out that its proof can be reduced to the preceding Lemma 4.2.

### Lemma 4.3

Suppose that $p> q+1 > 2$ and that *u* is a weak solution to (1.2), where assumption (1.3) holds true. Moreover, we consider a cylinder $Q_{2\varrho }^{(\lambda ,\theta )}(z_o) \Subset \Omega _T$ and assume that (3.1) together with either (4.1) or (4.2) is satisfied. Then the following Sobolev–Poincaré inequality holds:where $\max \left\{ \frac{p-1}{p},\frac{n}{n+2} \right\} \le \nu \le 1$ and $a = (u)_{z_o;\varrho }^{(\theta ,\lambda )}$. The preceding estimate holds for an arbitrary $\varepsilon \in (0,1)$ with a constant $c = c(n,p,q,C_1,C_\theta ,C_\lambda )>0$ and $\beta = \beta (n,p,q)>0$.

### Proof

Observe that $p> q+1 > 2$. Applying Lemma 2.2 and Hölder’s inequality with exponents $\frac{q+1}{q-1}$ and $\frac{q+1}{2}$, we estimateBy using Hölder’s inequality, $\theta$-subintrinsic scaling (4.1)_1_ for the first term and using Young’s inequality with exponents $\frac{p}{p-2}$ and $\frac{p}{2}$ we further obtainThe claim follows by using Lemma 4.2 for the latter term. $\square$

## Reverse Hölder inequality

In the next lemma, we combine the energy estimate (2.1) with the Sobolev–Poincaré inequalities from the preceding sections to prove a reverse Hölder inequality that will be a crucial tool for the proof of the higher integrability.

### Lemma 5.1

Let $q > 1$, $p > \frac{n(q+1)}{n+q+1}$ and *u* be a weak solution to (1.2) in the sense of Definition 1.1 and let $Q_{2\varrho }^{(\lambda ,\theta )}(z_o) \Subset \Omega _T$ be a cylinder for some $\varrho > 0$, $\lambda >0$ and $\theta > 0$. If (3.1) together with (3.2) or (3.3) is satisfied, then the following reverse Hölder inequality holds truefor $\max \left\{ \frac{p-1}{p}, \frac{n}{n+2},\frac{n}{n+2} \left( 1+\frac{2}{p}- \frac{2}{q} \right) , \frac{n(q+1)}{p(n+q+1)} \right\} \le \nu \le 1$ and a constant $c > 0$ depending on $n,p,q,C_o, C_1, C_\lambda , C_\theta$.

### Proof

We omit the center point $z_o$ from the notation for simplicity. Let $\varrho \le r < s \le 2\varrho$ and denote $a_\sigma = (u)_\sigma ^{(\lambda ,\theta )}$ for $\sigma \in \{r,s\}$. Lemma 2.6 impliesby using also Lemma 2.4 and denoting ${\mathcal {R}}_{r,s} = \frac{s}{s-r}$. We apply Lemma 3.3 for I and Lemma 3.1 for II if $q+1 \ge p$, and Lemmas 4.2 and 4.3, respectively, if $p > q+1$, which yieldsfor every $\varepsilon \in (0,1)$. We fix $\varepsilon = \frac{1}{2 c {\mathcal {R}}_{r,s}^{p^\sharp }}$, and use Lemma 2.1 to conclude the result. $\square$

We end this section with a technical lemma that will be needed to prove the $\theta$-singular scaling (3.3) in the cases in which the $\theta$-intrinsic scaling (3.2) is not available, see Sect. 6.4.

### Lemma 5.2

Let $q > 1$, $p > \frac{n(q+1)}{n+q+1}$ and *u* be a weak solution to (1.2) in the sense of Definition 1.1 and let $Q_{2\varrho }^{(\lambda ,\theta )}(z_o) \Subset \Omega _T$ be a cylinder for some $\varrho >0$, $\lambda >0$ and $\theta > 0$. If (3.1)_1_ and (3.2) with $C_\theta = 1$ are satisfied, we havefor $c = c(n,p,q,C_o,C_1,C_\lambda ) > 0$.

### Proof

We apply first (3.2)_2_ with $C_\theta = 1$, then the triangle inequality and Lemma 2.4, and finally, the triangle inequality again. In this way, we getHere we used the abbreviations ${\widehat{B}}=B_{{\hat{\varrho }}}^{(\theta )}$ and ${\widehat{Q}}:={\widehat{B}}\times \Lambda _\varrho ^{(\lambda )}$, with the radius ${\hat{\varrho }}\in [\frac{\varrho }{2},\varrho ]$ provided by Lemma 2.7. Observe that by Hölder’s inequalityBy Lemmas 2.3, 2.4 and 2.7, we obtainin which $c_\varepsilon$ depends on $\varepsilon , n,p,q,C_1$ and $C_\lambda$. On the last line, we also used (3.1)_1_ and Young’s inequality with exponents $\frac{2q}{q+1}$ and $\frac{2q}{q-1}$.

For the estimate of $\textrm{I}$, we consider the case $p > q+1$ first. In this case, Lemmas 2.4 and 4.1 implyfor $c = c(n,p,q,C_\lambda )$. Then, let us consider the case $q+1 \ge p$. By using Lemma 3.2 with $a = 0$, we have5.1for $c = c(n,p,q,C_\lambda )$. By using the energy estimate from Lemma 2.6 with $a= 0$, we obtainfor $c = c(p,q,C_o,C_1,C_\lambda )$, where we also used (3.2)_1_ and (3.1)_1_. By plugging this into (5.1), observing that $\frac{2(q+1)+ p(p-2)}{2(q+1) +p(q-1)} + p \frac{q+1-p}{2(q+1) + p(q-1)} = 1$, we use Young’s inequality to the first two terms including $\theta$ to conclude

$$\begin{aligned} \textrm{I} \le \varepsilon \theta ^\frac{2}{q+1} + c_\varepsilon \lambda ^\frac{2}{q+1}, \end{aligned}$$

in which $c_\varepsilon$ depends on $\varepsilon , n,p,q,C_o,C_1$ and $C_\lambda$. Collecting the estimates, we obtain in any caseBy choosing $\varepsilon = \frac{1}{6}$, the claim follows.

$$\square $$

## Proof of the higher integrability

This section is devoted to the proof of our main result, Theorem 1.2. Fix $Q_{4R}$ with $R > 0$ such that $Q_{8R} \Subset \Omega _T$ and6.1where the parameter $d\ge 1$ is defined in (1.4). Note that we can rewrite it as

$$\begin{aligned} d=\frac{p(q+1)}{(q+1)^2+(p^\sharp +n)(p-p^\sharp )}. \end{aligned}$$

Fix $$\lambda \ge \lambda _o$$ and

6.2 $$\begin{aligned} R_o=\min \left\{ \lambda ^\frac{p-2}{q+1},\lambda ^\frac{q-1}{q+1}\right\} R=\lambda ^{\frac{p+q-1-p^\sharp }{q+1}}R. \end{aligned}$$

Note that $R_o$ might be larger than *R* for certain values of parameters, but by definition of $Q_{2\varrho }^{(\lambda ,\theta )}(z_o)$, we still have the inclusion

$$\begin{aligned} Q_{2\varrho }^{(\lambda ,\theta )}(z_o) \subset Q_{2R}(z_o)\subset Q_{4R} \end{aligned}$$

for every $z_o \in Q_{2R}$, $\theta \ge \lambda$ and $\varrho \le R_o$.

The crucial step of the proof is to construct a suitable family of parabolic cylinders, which satisfy a Vitali type covering property and for which (3.1) and either  (3.2) or (3.3) hold true, so that the reverse Hölder inequality from Lemma 5.1 is applicable.

### Construction of a non-uniform system of cylinders

For fixed $z_o \in Q_{2R}$, $\lambda \ge \lambda _o$, and $\varrho \in (0,R_o]$, we define

$$\begin{aligned} {\tilde{\theta }}^{(\lambda )}_{z_o; \varrho }:= \inf \left\{ \theta \in [\lambda ,\infty ): \frac{1}{|Q_\varrho |} \iint _{Q_\varrho ^{(\lambda ,\theta )}(z_o)} \frac{|u|^{p^\sharp }}{\varrho ^{p^\sharp }}\, \,\! \textrm{d}x\,\! \textrm{d}t \le \lambda ^{2-p} \theta ^\frac{2p^\sharp + n (1-q)}{1+q} \right\} . \end{aligned}$$

Observe that the integral above converges to zero when $\theta \rightarrow \infty$, while the right-hand side blows up with speed $\theta ^\frac{2p^\sharp + n (1-q)}{1+q}$ provided that $q < \frac{n+2}{n-2}$ if $p \le q+1$, and $p > \frac{n}{2}(q-1)$ if $p > q+1$. Thus, there exists a unique ${\tilde{\theta }}^{(\lambda )}_{z_o; \varrho }$ for fixed $z_o,\varrho$ and $\lambda$ satisfying the above conditions. In case $\lambda$ and $z_o$ are clear from the context, we omit them from the notation.

By definition, one of the following two alternatives occurs; eitheror6.3Note that if ${\tilde{\theta }}_{R_o} > \lambda$, it follows from (6.1) that6.4In the last estimate, we distinguished between the cases $p\ge q+1$ and $\frac{n(q+1)}{n+q+1}<p<q+1$ and used the fact $\lambda \ge \lambda _o$.

The mapping $(0,R_o] \ni \varrho \mapsto {\tilde{\theta }}_\varrho$ is continuous by a similar argument as in [7] (see also [5, 6, 8]), but it is not non-increasing in general. Therefore, we define

$$\begin{aligned} \theta _{z_o; \varrho }^{(\lambda )}:= \max _{r\in [\varrho , R_o]} {\tilde{\theta }}_{z_o;r}^{(\lambda )}, \end{aligned}$$

which is clearly continuous (since ${\tilde{\theta }}_\varrho$ is) and non-increasing with respect to $\varrho$. Furthermore, let

$$\begin{aligned} {\tilde{\varrho }}:= {\left\{ \begin{array}{ll} R_o, &{}\text {if } \theta _\varrho = \lambda , \\ \inf \{s \in [\varrho ,R_o]: \theta _s = {\tilde{\theta }}_s \}, &{}\text {if } \theta _\varrho > \lambda . \end{array}\right. } \end{aligned}$$

Observe that $\theta _r = {\tilde{\theta }}_{{\tilde{\varrho }}}$ for every $r \in [\varrho ,{\tilde{\varrho }}]$. The following lemma summarizes some basic properties of the parameter $\theta _\varrho$.

#### Lemma 6.1

Let $\theta _\varrho$ be constructed as above. Then we have

(i)
(ii) $$\theta _\varrho \le \left( \frac{s}{\varrho } \right) ^\frac{(q+1)(n+ p^\sharp +q+ 1)}{2 p^\sharp + n(1-q)} \theta _s \quad \text {for every } 0< \varrho \le s \le R_o$$,
(iii) $$\theta _\varrho \le \left( \frac{4 R_o}{\varrho } \right) ^\frac{(q+1)(n+ p^\sharp +q+ 1)}{2 p^\sharp + n(1-q)} \lambda \quad \text {for every } 0 < \varrho \le R_o$$.

#### Proof

(i): Clearly, ${\tilde{\theta }}_s \le \theta _s \le \theta _\varrho$, which implies $Q_s^{(\lambda ,\theta _\varrho )} \subset Q_s^{(\lambda ,{\tilde{\theta }}_s)}$. Thus,where we have used the fact $2p^\sharp + n (1-q)>0$ that follows from the assumption $q<\max \{\frac{n+2}{n-2},\frac{2p}{n}+1\}$.

(ii): If $\theta _\varrho = \lambda$, the claim clearly holds. Suppose that $\lambda < \theta _\varrho$ and $s \in [{\tilde{\varrho }},R_o]$. We have

$$\begin{aligned} \theta _\varrho ^\frac{2 p^\sharp + n(1-q)}{q+1}&= {\tilde{\theta }}_{{\tilde{\varrho }}}^\frac{2 p^\sharp + n(1-q)}{q+1} = \frac{\lambda ^{p-2}}{|Q_{{\tilde{\varrho }}}|} \iint _{Q_{{\tilde{\varrho }}}^{(\lambda , \theta _{{\tilde{\varrho }}})}} \frac{|u|^{p^\sharp }}{{\tilde{\varrho }}^{p^\sharp }} \, \,\! \textrm{d}x \,\! \textrm{d}t \\&\le \left( \frac{s}{{\tilde{\varrho }}} \right) ^{n+ p^\sharp + q +1} \frac{\lambda ^{p-2}}{|Q_{s}|} \iint _{Q_s^{(\lambda ,\theta _s)}} \frac{|u|^{p^\sharp }}{s^{p^\sharp }} \, \,\! \textrm{d}x \,\! \textrm{d}t \\&\le \left( \frac{s}{{\tilde{\varrho }}} \right) ^{n+ p^\sharp + q +1} \theta _s^\frac{2 p^\sharp + n(1-q)}{q+1}, \end{aligned}$$

which implies the claim. If $s \in [\varrho , {\tilde{\varrho }})$, then $\theta _\varrho = \theta _s$ and the claim clearly holds.

(iii): By choosing $s = R_o$ in (ii), and using (6.4) (observe that $\theta _{R_o} = {\tilde{\theta }}_{R_o}$), we have

$$\begin{aligned} \theta _\varrho \le \left( \frac{R_o}{\varrho } \right) ^\frac{(q+1)(n+ p^\sharp +q+ 1)}{2 p^\sharp + n(1-q)} \theta _{R_o} \le \left( \frac{4 R_o}{\varrho } \right) ^\frac{(q+1)(n+ p^\sharp +q+ 1)}{2 p^\sharp + n(1-q)} \lambda , \end{aligned}$$

completing the proof. $\square$

### Vitali-type covering property

#### Lemma 6.2

Let $\lambda \ge \lambda _o$. There exists ${{\hat{c}}} = {{\hat{c}}} (n,p,q) \ge 20$ such that the following holds: Let ${\mathcal {F}}$ be any collection of cylinders $Q_{4r}^{(\lambda ,\theta _{z;r}^{(\lambda )})} (z)$, where $Q_{r}^{(\lambda ,\theta _{z;r}^{(\lambda )})} (z)$ is a cylinder of the form that is constructed in Sect. 6.1 with radius $r \in \left( 0,\frac{R_o}{{{\hat{c}}}}\right)$. Then, there exists a countable, disjoint subcollection ${\mathcal {G}}$ of ${\mathcal {F}}$ such that

$$\begin{aligned} \bigcup _{Q \in {\mathcal {F}}} Q \subset \bigcup _{Q \in {\mathcal {G}}} {\widehat{Q}}, \end{aligned}$$

where $\widehat{Q}$ denotes the $\frac{1}{4} {{\hat{c}}}$-times enlarged *Q*, i.e., if $Q = Q_{4r}^{(\lambda ,\theta _{z;r}^{(\lambda )})} (z)$, then ${\widehat{Q}} = Q_{{{\hat{c}}} r}^{(\lambda ,\theta _{z;r}^{(\lambda )})} (z)$.

#### Proof

As in [7] (see also [5, 6, 8]), consider

$$\begin{aligned} {\mathcal {F}}_j:= \left\{ Q_{4r}^{(\lambda ,\theta _{z;r}^{(\lambda )})} (z) \in {\mathcal {F}}: \tfrac{R_o}{2^j {{\hat{c}}}} < r \le \tfrac{R_o}{2^{j-1} {{\hat{c}}}} \right\} , \quad j \in {\mathbb {N}}. \end{aligned}$$

Let ${\mathcal {G}}_1$ be a maximal disjoint subcollection of ${\mathcal {F}}_1$, which is finite by Lemma 6.1 (iii). At stage $k \in {\mathbb {N}}_{\ge 2}$, let ${\mathcal {G}}_k$ be a maximal disjoint collection of cylinders in

$$\begin{aligned} \left\{ Q\in {\mathcal {F}}_k: Q \cap Q^* = \varnothing \text { for any } Q^* \in \bigcup _{j=1}^{k-1} {\mathcal {G}}_j \right\} , \end{aligned}$$

and define

$$\begin{aligned} {\mathcal {G}} = \bigcup _{j=1}^{\infty } {\mathcal {G}}_j, \end{aligned}$$

which is countable since ${\mathcal {G}}_j$ for every $j \in {\mathbb {N}}$ is finite.

Our objective to show is that for every $Q \in {\mathcal {F}}$ there exists $Q^* \in {\mathcal {G}}$ such that $Q \cap Q^* \ne \varnothing$ and $Q \subset {\widehat{Q}}^*$. To this end, let $Q = Q_{4r}^{(\lambda ,\theta _{z;r}^{(\lambda )})} (z) \in {\mathcal {F}}$, which implies that there exists $j \in {\mathbb {N}}$ such that $Q \in {\mathcal {F}}_j$. By maximality of ${\mathcal {G}}_j$, there exists $Q^* = Q_{4r_*}^{(\lambda ,\theta _{z_*;r_*}^{(\lambda )})} (z_*) \in \bigcup _{i=1}^j {\mathcal {G}}_i$ such that $Q \cap Q^* \ne \varnothing$. By definitions of ${\mathcal {F}}_j$ and ${\mathcal {G}}_j$, it follows that $r < 2 r_*$. This immediately implies

6.5 $$\begin{aligned} \Lambda _{4r}^{(\lambda )}(t) \subset \Lambda _{12 r_*}^{(\lambda )}(t_*). \end{aligned}$$

Let ${{\tilde{r}}}_* \in [r_*,R_o]$ be defined as in the earlier section. It follows that

6.6 $$\begin{aligned} \Lambda _{4 {{\tilde{r}}}_*}^{(\lambda )}(t_*) \subset \Lambda ^{(\lambda )}_{10 {{\tilde{r}}}_*}(t). \end{aligned}$$

Next we show that

6.7 $$\begin{aligned} \theta _{z_*;r_*}^{(\lambda )} \le 64^\frac{(q+1)(n+p^\sharp +q+1)}{2p^\sharp + n(1-q)} \theta _{z;r}^{(\lambda )}. \end{aligned}$$

Observe that if $\theta _{z_*,r_*}^{(\lambda )} = \lambda$ (which implies ${{\tilde{r}}}_* = R_o$), we have

$$\begin{aligned} \theta _{z_*;r_*}^{(\lambda )} = \lambda \le \theta _{z;r}^{(\lambda )}. \end{aligned}$$

On the other hand, if $\lambda < \theta _{z_*;r_*}^{(\lambda )} (= \theta _{z_*;{{\tilde{r}}}_*}^{(\lambda )} = {\tilde{\theta }}_{z_*;{{\tilde{r}}}_*}^{(\lambda )})$, we have by (6.3) that

6.8 $$\begin{aligned} ( \theta _{z_*;r_*}^{(\lambda )})^\frac{2 p^\sharp + n(1-q)}{q+1} = \frac{\lambda ^{p-2}}{|Q_{{{\tilde{r}}}_*}|} \iint _{Q_{\tilde{r}_*}^{(\lambda , \theta _{z_*;r_*}^{(\lambda )})} (z_*)} \frac{|u|^{p^\sharp }}{{{\tilde{r}}}_*^{p^\sharp }} \, \,\! \textrm{d}x \,\! \textrm{d}t. \end{aligned}$$

Fix $\eta = 16$. By distinguishing between the cases ${{\tilde{r}}}_* \le \frac{R_o}{\eta }$ and ${{\tilde{r}}}_* > \frac{R_o}{\eta }$, for the latter we obtainsimilarly as in (6.4), since $\lambda \le \theta _{z;r}^{(\lambda )}$. For the former case, we may assume that $\theta _{z_*;r_*}^{(\lambda )} \ge \theta _{z;r}^{(\lambda )}$ since otherwise (6.7) clearly holds. Furthermore, observe that $r \le 2 r_* \le 2 {{\tilde{r}}}_* \le \eta {{\tilde{r}}}_*$, which implies

$$\begin{aligned} \theta _{z_*;r_*}^{(\lambda )} \ge \theta _{z;r}^{(\lambda )} \ge \theta _{z;\eta {{\tilde{r}}}_*}^{(\lambda )}. \end{aligned}$$

Thus, we have

$$\begin{aligned} B_{4 {{\tilde{r}}}_*}^{(\theta _{z_*,r_*}^{(\lambda )})} (x_*) \subset B_{\eta {{\tilde{r}}}_*}^{(\theta _{z, \eta {{\tilde{r}}}_*}^{(\lambda )})} (x). \end{aligned}$$

Using this together with (6.6) to estimate the right-hand side of (6.8) from above, we deduce

$$\begin{aligned} ( \theta _{z_*;r_*}^{(\lambda )})^\frac{2 p^\sharp + n(1-q)}{q+1}&\le \frac{ \eta ^{p^\sharp }\lambda ^{p-2}}{|Q_{{{\tilde{r}}}_*}|} \iint _{Q_{\eta {{\tilde{r}}}_*}^{(\lambda , \theta _{z,\eta {{\tilde{r}}}_*}^{(\lambda )})} (z)} \frac{|u|^{p^\sharp }}{ (\eta {{\tilde{r}}}_*)^{p^\sharp }} \, \,\! \textrm{d}x \,\! \textrm{d}t \\&\le \eta ^{n+p^\sharp +q+1} (\theta _{z;r}^{(\lambda )})^\frac{2 p^\sharp + n(1-q)}{q+1}, \end{aligned}$$

where we used Lemma 6.1 (i) with $\varrho =s=\eta {{\tilde{r}}}_*$ for the last estimate. Therefore, we have shown that (6.7) holds in every case. By choosing

$$\begin{aligned} {{\hat{c}}} \ge 4 \big (4\cdot 64^\frac{(q-1)(n+p^\sharp +q+1)}{2p^\sharp + n(1-q)}+1\big ) \ge 20, \end{aligned}$$

it follows that $B_{4r}^{(\theta _{z;r})}(x) \subset B_{{{\hat{c}}} r_*}^{(\theta _{z_*;r_*})}(x_*)$. This is due to the fact that for every $x_1\in B_{4r}^{(\theta _{z;r})}(x)$ we have

$$\begin{aligned} |x_1 - x_*|&\le |x_1 - x| + |x - x_*| \le 2 \theta _{z;r}^\frac{1-q}{1+q} (4r) + \theta _{z_*;r_*}^\frac{1-q}{1+q} (4r_*) \\ {}&\le 4 \theta _{z_*;r_*}^\frac{1-q}{1+q} r_* \left( 4\cdot 64^\frac{(q-1)(n+p^\sharp +q+1)}{2p^\sharp + n(1-q)} + 1 \right) \le {{\hat{c}}} \theta _{z_*;r_*}^\frac{1-q}{1+q} r_*,\end{aligned}$$

where we used $Q \cap Q^*\ne \varnothing$, $r < 2r_*$ and (6.7). By also recalling (6.5), we have

$$\begin{aligned} Q = Q_{4r}^{(\lambda ,\theta _{z;r}^{(\lambda )})} (z) \subset {\widehat{Q}}^* = Q_{{{\hat{c}}} r_*}^{(\lambda ,\theta _{z_*;r_*}^{(\lambda )})} (z_*), \end{aligned}$$

which completes the proof. $\square$

### Stopping time argument

Let6.9Consider $\lambda > \lambda _o$ and $r\in (0,2R]$ and define

$$\begin{aligned} {\textbf{E}}(r,\lambda ):= \big \{ z \in Q_r: z \text { is a Lebesgue point of } |Du| \text { and } |Du|(z)> \lambda \big \}, \end{aligned}$$

in which Lebesgue points are understood in context of cylinders of the type $Q_{\varrho }^{(\lambda ,\theta _{\varrho })}$ constructed in Sect. 6.1.

Consider radii $R \le R_1 < R_2 \le 2 R$ and concentric cylinders $Q_R \subset Q_{R_1} \subset Q_{R_2} \subset Q_{2R}$. Fix $z_o \in {\textbf{E}} (R_1,\lambda )$ and denote $\theta _s = \theta ^{(\lambda )}_{z_o; s}$ for $s \in (0,R_o]$. By definition of ${\textbf{E}}(R_1,\lambda )$, we have6.10Let ${{\hat{c}}}$ denote the constant from the Vitali type covering lemma, Lemma 6.2, and consider

6.11 $$\begin{aligned} \lambda> B \lambda _o, \quad \text {where}\quad B:= \left( \frac{4 {{\hat{c}}} R}{R_2-R_1} \right) ^\frac{d p^\sharp (n+2)(q+1)}{p (2p^\sharp + n(1-q))} >1. \end{aligned}$$

Let $\frac{R_2-R_1}{{\mathfrak {m}}} \le s \le R_o$, where ${\mathfrak {m}} = {{\hat{c}}} \lambda ^\frac{p^\sharp +1 - p-q}{q+1}$. By (6.9), Lemma 6.1 (iii) and (6.2) we haveBy the above estimate, (6.10) and the continuity of the integral (w.r.t. *s*) there exists a maximal radius $\varrho _{z_o} \in (0,\frac{R_2-R_1}{{\mathfrak {m}}})$ such that6.12The maximality of the radius implies6.13By combining the last inequality with Lemma 6.1  (ii) and using the fact that $\varrho \mapsto \theta _\varrho$ is non-increasing, we have6.14for every $s \in (\varrho _{z_o}, R_o]$. Observe that also clearly $Q_{{{\hat{c}}} \varrho _{z_o}}^{(\lambda ,\theta _{\varrho _{z_o}})} (z_o) \subset Q_{R_2}$.

### A reverse Hölder inequality

Fix $z_o \in {\textbf{E}} (R_1,\lambda )$ and $\lambda > B \lambda _o$ as defined in (6.11). We will show that6.15for exponents $\max \left\{ \frac{n(q+1)}{p(n+q+1)}, \frac{p-1}{p}, \frac{n}{n+2},\frac{n}{n+2} \left( 1+\frac{2}{p}- \frac{2}{q} \right) \right\} \le \nu \le 1$ and a constant $c = c(n,p,q,C_o,C_1) > 0$.

First, we consider the case ${\tilde{\varrho }}_{z_o} \le 2 \varrho _{z_o}$. Observe that this implies ${\tilde{\varrho }}_{z_o}<R_o$, and therefore $\lambda < \theta _{\varrho _{z_o}} = \theta _{{\tilde{\varrho }}_{z_o}} = {\tilde{\theta }}_{{\tilde{\varrho }}_{z_o}}$. By Lemma 6.1 (i) with $s = 2 {\tilde{\varrho }}_{z_o}$ and (6.3) we havei.e., condition (3.2) holds with $C_\theta = 1$ and $\varrho ={\tilde{\varrho }}_{z_o}$. By (6.14) and (6.12), we deducewhich implies that also (3.1) holds with $C_\lambda = C_\lambda (n,p,q)$. Thus, we can use Lemma 5.1 to obtainfor $c = c(n,p,q,C_o,C_1)$. This proves (6.15) in the first case.

Then, we consider the case ${\tilde{\varrho }}_{z_o} > 2 \varrho _{z_o}$. Observe that by (6.14) and (6.12) we havesuch that (3.1) holds with $C_\lambda = C_\lambda (n,p,q)$ and $\varrho =\varrho _{z_o}$. Furthermore, (3.3)_1_ with $C_\theta = 1$ holds by Lemma 6.1 (i). For the proof of (3.3)_2_, we first consider the case ${\tilde{\varrho }}_{z_o} \in \left[ \frac{R_o}{2},R_o\right]$. In this case, by Lemma 6.1 (iii) and (6.12) we havewhich implies (3.3)_2_ with $C_\lambda =C_\lambda (n,p,q)$. Now we are left with the case ${\tilde{\varrho }}_{z_o} \in (2 \varrho _{z_o},\frac{R_o}{2})$. Observe that since ${\tilde{\varrho }}_{z_o} < R_o$, it follows that $\lambda < \theta _{\varrho _{z_o}} = \theta _{{\tilde{\varrho }}_{z_o}} = \tilde{\theta }_{{\tilde{\varrho }}_{z_o}}$ by definition so that Lemma 6.1 (i) and (6.3) implyFurthermore, by $\theta _{\varrho _{z_o}} = \theta _{{\tilde{\varrho }}_{z_o}}$, the monotonicity of $\varrho \mapsto \theta _\varrho$, Lemma 6.1 (ii) and (6.13) we obtainThus, $Q_{{\tilde{\varrho }}_{z_o}}^{(\lambda , \theta _{\varrho _{z_o}})}(z_o)$ is $\theta$-intrinsic (with $C_\theta = 1$) and $\lambda$-subintrinsic. We use Lemmas 5.2 and 6.1 (i) (observe that ${\tilde{\varrho }}_{z_o} / 2 > \varrho _{z_o}$) to obtainThus, by (6.12)holds true, which implies (3.3)_2_ with $C_\theta =C_\theta (n,p,q,C_o,C_1)$ also in this final case. Therefore, we have established that (3.1) and (3.3) hold true with $\varrho =\varrho _{z_o}$ in the case ${\tilde{\varrho }}_{z_o}>2\varrho _{z_o}$. This enables us to use Lemma 5.1 to conclude that (6.15) holds in any case.

### Final argument

The rest of the proof is identical to [7, Sect. 6.5 & 6.6]. Hence, we refrain ourselves from repeating the computations and only sketch the final argument.

We have that if $\lambda$ satisfies (6.11), then for every $z_o\in {\textbf{E}}(R_1,\lambda )$ there exists a cylinder $Q_{\varrho _{z_o}}^{(\lambda ,\theta _{z_o;\varrho _{z_o}})} (z_o)$ in which (6.12), (6.13), (6.14) and (6.15) hold true and Lemma 6.2 is satisfied. Furthermore, $Q_{{{\hat{c}}}\varrho _{z_o}}^{(\lambda ,\theta _{z_o;\varrho _{z_o}})} (z_o) \subset Q_{R_2}$ in which ${{\hat{c}}}$ is the constant from Lemma 6.2.

By denoting

$$\begin{aligned} {\textbf{F}}(r,\lambda ):= \left\{ z \in Q_{r}: z \text { is a Lebesgue point of } |F| \text { and } |F|(z) > \lambda \right\} , \end{aligned}$$

we deduce as in [7, Sect. 6.5] that

$$\begin{aligned} \iint _{{\textbf{E}}(R_1,{\tilde{\lambda }})} |Du|^p \, \,\! \textrm{d}x \,\! \textrm{d}t \le c \iint _{{\textbf{E}}(R_2,{\tilde{\lambda }})} {\tilde{\lambda }}^{(1-\nu )p} |Du|^{\nu p} \, \,\! \textrm{d}x \,\! \textrm{d}t +c \iint _{{\textbf{F}}(R_2,{\tilde{\lambda }})} |F|^p \, \,\! \textrm{d}x \,\! \textrm{d}t \end{aligned}$$

for every ${\tilde{\lambda }} \ge \eta B \lambda _o$, in which $\eta = \eta (n,p,q,C_o,C_1) \in (0,1]$ and *B* and $\lambda _o$ are defined in (6.11) and (6.9).

By a truncation and Fubini type argument, the estimate in Theorem 1.2 can be deduced exactly as in [7, Sect. 6.6].
